# Post-intensive care syndrome in out-of-hospital cardiac arrest patients: A prospective observational cohort study

**DOI:** 10.1371/journal.pone.0276011

**Published:** 2022-10-14

**Authors:** Alessia Vincent, Katharina Beck, Emanuel Thommen, Madlaina Widmer, Christoph Becker, Nina Loretz, Sebastian Gross, Jonas Mueller, Simon A. Amacher, Chantal Bohren, Rainer Schaefert, Jens Gaab, Stephan Marsch, Christian Emsden, Kai Tisljar, Raoul Sutter, Sabina Hunziker

**Affiliations:** 1 Medical Communication and Psychosomatic Medicine, University Hospital Basel, Klingelbergstrasse, Basel, Switzerland; 2 Division of Clinical Psychology and Psychotherapy, Faculty of Psychology, University of Basel, Missionsstrasse, Basel, Switzerland; 3 Emergency Department, University Hospital Basel, Petersgraben, Basel, Switzerland; 4 Department of Intensive Care, University Hospital Basel, Petersgraben, Basel, Switzerland; 5 Medical Faculty, University of Basel, Klingelbergstrasse, Basel, Switzerland; University Medical Center Goettingen, GERMANY

## Abstract

**Introduction:**

Intensive care unit patients are at risk for post-intensive care syndrome (PICS), which includes psychological, physical and/or cognitive sequelae after their hospital stay. Our aim was to investigate PICS in adult patients with out-of-hospital cardiac arrest (OHCA).

**Methods:**

In this prospective observational cohort study, we assessed risks for PICS at 3 and 12-month follow-up within the following domains: a) physical impairment (EuroQol [EQ-5D-3L]), b) cognitive functioning (Cerebral Performance Category [CPC] score >1, modified Rankin Scale [mRS] >2) and c) psychological burden (Hospital Anxiety and Depression Scale [HADS], Impact of Event Scale-Revised [IES-R]).

**Results:**

At 3 months, 69/139 patients (50%) met the definition of PICS including 37% in the physical domain, 25% in the cognitive domain and 13% in the psychological domain. Intubation (OR 2.3, 95%CI 1.1 to 5,0 p = 0.03), sedatives (OR 3.4, 95%CI 1 to 11, p = 0.045), mRS at discharge (OR 4.3, 95%CI 1.70 to 11.01, p = 0.002), CPC at discharge (OR 3.3, 95%CI 1.4 to 7.6, p = 0.005) and post-discharge work loss (OR 13.4, 95%CI 1.7 to 107.5, p = 0.014) were significantly associated with PICS. At 12 months, 52/110 (47%) patients had PICS, which was associated with prolonged duration of rehabilitation, higher APACHE scores, and higher mRS and CPC scores at hospital discharge.

**Conclusions:**

Nearly half of long-term OHCA survivors show PICS after 3 and 12 months. These high numbers call for more emphasis on appropriate screening and treatment in this patient population. Future studies should evaluate whether early identification of these patients enables preventive strategies and treatment options.

## Introduction

Out-of-hospital cardiac arrest (OHCA) remains an important cause of death worldwide [1]. Less than a quarter of OHCA patients survive to hospital admission, and only half of initial survivors are discharged from the hospital alive [2]. Although therapeutic advances in intensive care medicine result in a higher number of ICU survivors, the overall ICU mortality decreased only very slightly over time due to the steadily increase of patients’ age and the number of comorbidities upon ICU admission [3]. Also, the risk of severe neurological deficits in ICU patients remains high [4,5] particularly in survivors of an out-of-hospital cardiac arrest (OHCA). In consequence, long-term physical, neurological and mental health status of ICU survivors has become an increasing concern in recent years [6]. These long-term impairments have been summarized under the term post-intensive care syndrome (PICS), which is commonly defined as new or aggravated dysfunction(s) in the physical, cognitive and/or mental (psychiatric) domain after critical illness [7]. Several studies suggest that more than 50% of ICU survivors suffer from at least one component of PICS [6,8]. Accordingly, PICS is becoming a more widely used concept in current clinical practice, even though attempts to define it with clinical accuracy are still ongoing [9].

Importantly, there is insufficient research data regarding the risk for PICS in the population of OHCA patients, although this population of patients is clearly at increased risk to suffer from long-term impairments and have worse physical and social functioning compared to the general population [10]. Studies have suggested that a relevant number of OHCA patients have moderate disabilities, poor autonomy and cognitive impairments particularly in regard to memory, attention and executive functioning [10–14]. In addition, OHCA patients are at increased risk for symptoms of depression, anxiety and posttraumatic stress disorder (PTSD) [15,16]. There are several well-known risk factors for adverse long-term health after OHCA including low-flow time, clinical severity at ICU admission, prolonged coma duration, and mechanical ventilation [11]. Also, young age and female gender was associated with higher risk for poor health [12].

Yet, to the best of our knowledge, only few studies have addressed the concept of PICS in OHCA patients. Better understanding the risk of PICS in OHCA patients is important for adequate future screening and treatment of patients at risk and may help to prevent PICS. Herein, we investigated the prevalence and potential risk factors for PICS in a well-defined cohort of adult OHCA survivors among the domains of physical, cognitive and psychological symptoms.

## Materials and methods

### Study setting

The COMMUNICATE study is an ongoing prospective observational cohort study (from 10/2012 to 10/2025) at ICU of the University Hospital Basel, a Swiss tertiary care hospital with ongoing sampling. The aim of the trial is to investigate the prognosis and long-term outcomes in consecutive adult patients after cardiac arrest. The methods applied in this study have been published previously [17–19]. The COMMUNICATE trial was approved by the local Ethics Committee (Ethics Committee Northwest and Central Switzerland, EKNZ; approval reference number: 2019–01162) and is conducted in accordance with the declaration of Helsinki. All patients, or in case of unconsciousness, patients’ next of kin provided written informed consent for study participation.

### Study population

We included adult patients after OHCA who were admitted to the ICU and who participated in the 3-month and/or 12-month follow-up assessment after hospital/ICU admission. Further, we also included patients with in-hospital cardiac arrest (IHCA) if these were not monitored and had thus a similar risk for adverse outcome compared to OHCA patients. No exclusion criteria regarding patient characteristics, e.g., consciousness, type, severity, or duration of cardiac arrest were used.

### Data collection

Data were prospectively collected upon ICU admission. Patients’ medical characteristics were extracted from hospital medical records. Further, we conducted predefined and structured telephone interviews with patients 3 and 12 months after ICU admission to evaluate outcomes. To assess outcomes, the research team performed systematic telephone interviews with patients lasting for around 20 minutes. Thereby, questionnaires were read aloud and patients’ answers were recorded.

### Measures

#### Baseline predictor variables

We calculated all clinical scores at ICU arrival as suggested in original publications [20,21]. From hospital medical records, we collected patients’ sociodemographic information (e.g., age, gender, working status), the setting of cardiac arrest (e.g., location, initial rhythm, no-flow time, low-flow time), adrenaline (epinephrine) dose given), the reason for OHCA (i.e., coronary heart disease, arrhythmogenic, other reason), the ICU treatment received (e.g., intubation, targeted temperature management, use of vasoactive or sedative medication), medical complications during ICU stay (e.g., delirium), comorbidities (e.g., smoking status, hyperlipidemia, coronary disease, diabetes, renal failure, malignant disease), and ICU and hospital length of stay. Further, we assessed the number of weeks in rehabilitation and working status three months after hospitalization.

#### Outcome measures

All outcome measures were assessed at 3-month and 12-month follow-up. The primary outcome PICS was defined as symptoms or impairment in at least one of the following domains, as previously defined [7]: physical impairment, cognitive impairment and/or psychological distress. The primary endpoint was PICS measured at 3 months and secondary endpoint was PICS at 12 months follow-up.

*Physical impairment*. Physical impairment was evaluated with the EuroQol questionnaire (EQ-5D-3L), an established, extensively validated, as well as time-efficient self-report measure which assesses general health-related quality of life [22]. The EQ-5D-3L comprises five dimensions, i.e., mobility, self-care, usual activities, pain/discomfort and anxiety/depression, which can be rated on three levels, i.e. no problems, some problems and extreme problems. These dimensions can be summarized in an index ranging from -0.5 “worse than death” to 1 “full health” [23]. We used a cut-off score of ≤0.8 to determine relevant physical impairment. Cronbach’s alpha for this sample was α = 0.65.

*Cognitive impairment*. To assess cognitive impairment, we used the Cerebral Performance Category (CPC) [24] and the modified Rankin Scale (mRS) [25], two expert-rated and time-efficient scales.

The CPC measures patients’ neurological status. It distinguishes five levels. In line with previous studies, we defined level 1 (good recovery) as favorable neurological outcome, and 2 (moderate disability), 3 (severe disability), 4 (vegetative state) and 5 (death) as poor neurological outcome [26].

The mRS scale assesses neurological function on a scale from 0 to 6. We defined levels 0 (no symptoms), 1 (no significant disability despite symptoms; able to carry out all usual duties and activities) and 2 (slight disability; unable to carry out all previous activities, but able to look after own affairs without assistance) as favorable outcome; and levels 3 (moderate disability; requiring some help, but able to walk without assistance) 4 (moderately severe disability, unable to walk and attend to bodily needs without assistance), 5 (severe disability; bedridden, incontinent and requiring constant nursing care and attention) and 6 (dead) were defined as unfavourable outcome [25,27].

*Psychological distress*. Psychological distress was defined as clinically relevant symptoms of anxiety, depression and/or PTSD. Symptoms of depression and anxiety were assessed with the Hospital Anxiety and Depression Scale (HADS) [28], a self-report instrument specifically developed for hospitalized patients with medical conditions. Good reliability and validity with a Cronbach’s alpha of 0.83 and 0.82 for the subscales anxiety and depression, respectively, has been demonstrated, as well as an optimal balance between sensitivity and specificity of approximately 0.80 when applying a cut-off score of ≥8 on both subscales [29]. Therefore, a score of ≥8 on the depression and/or anxiety subscale (range 0 to 21) of the HADS was considered as clinically relevant for the purpose of the study [28,29]. The HADS consists of 14 items and Cronbach’s alpha for this population was α = .84. PTSD symptoms were assessed by the Impact of Event Scale-Revised (IES-R) [30]. This self-report measure with 22 items covers three symptom domains, i.e., intrusion, avoidance, and hyperarousal. It shows high internal consistency with a Cronbach’s alpha of 0.96 and good diagnostic accuracy at a cut-off score of 1.5 [31], which we applied in this study. Cronbach’s alpha for this population was α = .92.

### Statistical analysis

Descriptive statistics, i.e., frequencies and percentages for binary and categorical variables, as well as medians and interquartile ranges for continuous variables were used to present sociodemographic and clinical characteristics of the study population. The primary endpoint was PICS defined as a physical, cognitive and/or psychological impairment measured with different scales as defined above (i.e., in one of the five outcome measures). To evaluate associations between potential risk factors and PICS at 3- and 12-month follow-up, logistic regression analyses were performed for the primary endpoint and separately for the three domains of PICS. As a measure of association, we report odds ratios (OR) and 95% confidence intervals (CI). In addition, univariable logistic regression analyses were adjusted for age and gender. We did not perform further multivariable analyses due to the low number of events to avoid overfitting. Further, a chi-square test and cross-tables were used to determine the persistence of patients with PICS between 3- and 12-month follow-up. Pearson correlations were calculated between the PICS domains physical, cognitive and psychological symptoms in a correlation matrix at 3 and 12 months. Stata 15 (StataCorp, College Station, Texas, USA) was used for all statistical analyses. Statistical significance was defined as a p-value of <0.05 (two-tailed).

## Results

### Study sample and baseline characteristics

**Fig 1:**
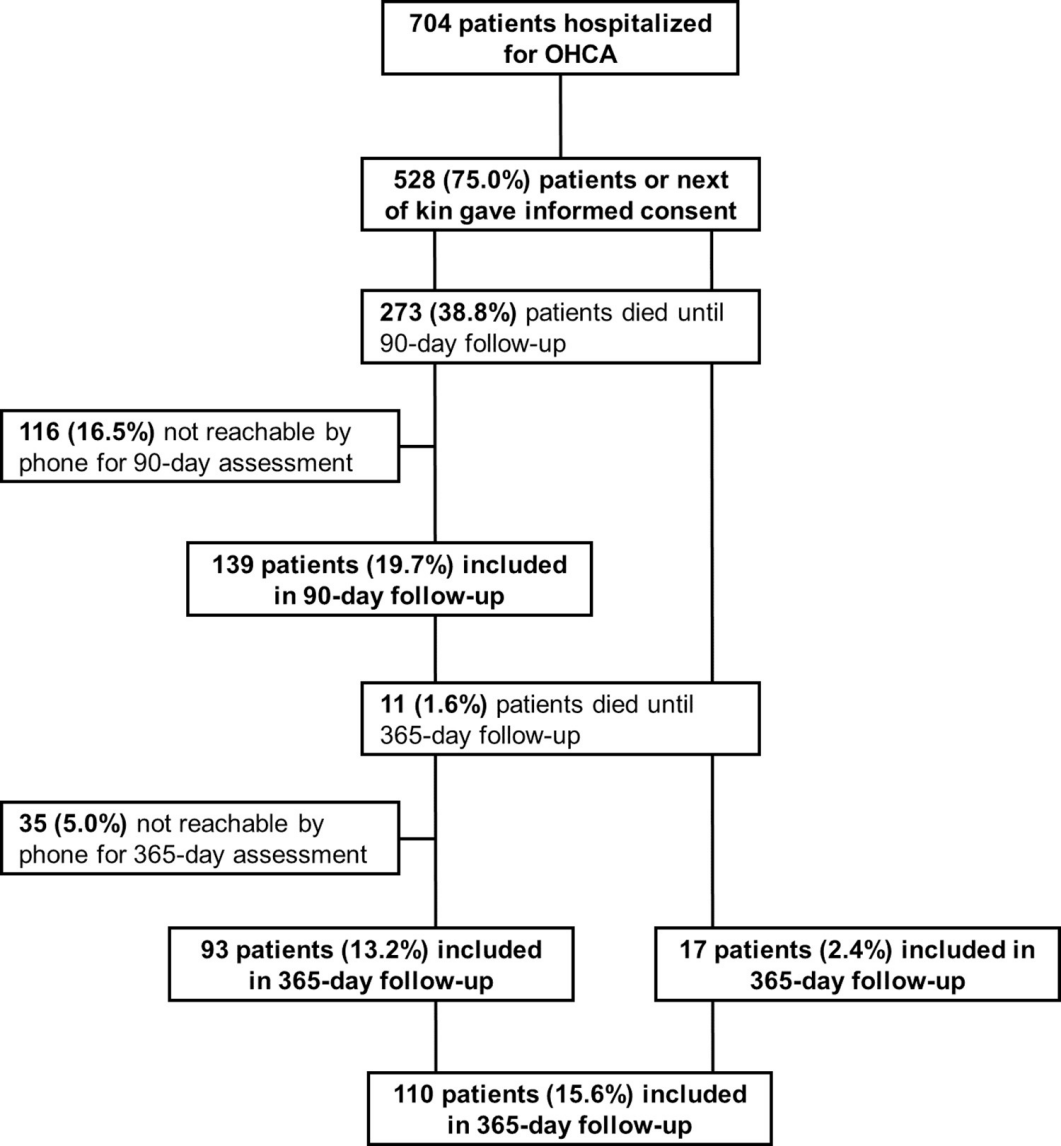
Flow diagram of the study population.

One-hundred fifty-six patients completed at least one of two follow-up interviews; 139 (89.1%) patients completed the 3-month interview, and 110 (70.5%) the 12-month interview. Ninety-three (59.6%) participants completed both interviews. Eleven (7.1%) patients died between the 3- and 12-month follow-up. A flow diagram of the study population is shown in **Fig 1**.

Sociodemographic and clinical characteristics of the study population and for patients included in the 3-month and 12-month follow-ups are shown in **Table 1**. Median age of patients was 62.8 years and 17% were female. The median duration of ICU stay was 4 days and median hospital length of stay was 13 days. Patients had a high burden of comorbidities and cardiovascular risk factors.

**Table 1:** Sociodemographic and clinical characteristics of the study population.

Factor		All patients	3 months	12 months
N		156	139	110
**Sociodemographics**				
Age, median (IQR)		62.8 (54, 73.2)	63.2 (54.3, 73.5)	62.6 (53.9, 73.2)
Female, n (%)		27 (17.3%)	22 (15.8%)	17 (15.5%)
In partnership, n (%)		123 (80.9%)	109 (80.7%)	90 (82.6%)
Children, n (%)		128 (82.1%)	114 (82.0%)	92 (83.6%)
Highest education	School, n (%)	14 (11.0%)	12 (10.8%)	12 (11.9%)
	Diploma/ apprenticeship, n (%)	90 (70.9%)	79 (71.2%)	72 (71.3%)
	University, n (%)	23 (18.1%)	20 (18.0%)	17 (16.8%)
Employed at baseline, n (%)		72 (48.3%)	61 (46.2%)	54 (50.0%)
**Setting of cardiac arrest**				
Setting of cardiac arrest	At home	43 (28.7%)	38 (28.6%)	31 (29.0%)
	In public	95 (63.3%)	84 (63.2%)	69 (64.5%)
	IHCA	12 (8.0%)	11 (8.3%)	7 (6.5%)
Observed cardiac arrest		143 (91.7%)	126 (90.6%)	106 (96.4%)
	Bystander CPR	124 (79.5%)	112 (80.6%)	83 (75.5%)
	Professional bystander	39 (48%)	37 (47%)	26 (57%)
Initial rhythm	Ventricular tachycardia	8 (5.2%)	5 (3.6%)	7 (6.4%)
	Ventricular fibrillation	114 (73.5%)	102 (73.9%)	81 (74.3%)
	Asystole	7 (4.5%)	6 (4.3%)	6 (5.5%)
	Pulseless electrical activity	9 (5.8%)	9 (6.5%)	7 (6.4%)
	Unknown	17 (11.0%)	16 (11.6%)	8 (7.3%)
No-flow (min), median (IQR)		0 (0, 2)	0 (0, 2)	0 (0, 2)
Low-flow (min), median (IQR)		11 (8, 20)	12 (9, 20)	11.5 (8, 20)
Time until ROSC (min), median (IQR)		15 (10, 23)	15 (10, 25)	15 (10, 25)
Adrenaline	No adrenaline	77 (55.0%)	70 (56.0%)	56 (57%)
	>0 and <3 mg	33 (23.6%)	26 (20.8%)	23 (23%)
	≥3 mg	30 (21.4%)	29 (23.2%)	19 (19%)
**Clinical scores at ICU arrival**				
APACHE II, median (IQR)		25 (19, 30)	25 (20, 30)	25 (19, 30)
SAPS II, median (IQR)		58 (45, 66)	58 (43, 66)	58 (45, 68)
GCS, median (IQR)		4 (3, 14)	4 (3, 14)	5 (3, 14)
**Days in ICU, median (IQR)**		4 (2, 7)	4 (2, 7)	4 (2, 7)
**Total days of hospital stay, median (IQR)**		13 (8, 18)	13 (8, 17)	13 (8, 19)

Note: Data are presented as n (%) or median (interquartile range). Abbreviations: APACHE II, Acute Physiology And Chronic Health Evaluation Score II; SAPS II, Simplified Acute Physiology Score II; GCS, Glasgow Coma Scale; OHCA, out-o-hospital cardiac arrest; IHCA, in-hospital cardiac arrest; IQR, interquartile range; CPR, cardiopulmonary resuscitation; ROSC, return of spontaneous circulation; IABP, intra-aortal balloon pump; mRS, modified Rankin Scale; CPC, Cerebral Performance Category.

### Primary endpoint: PICS 3 months after hospitalization

Of 139 patients, 69 patients (49.6%) showed evidence of PICS 3 months after OHCA. Of those, 36.7% showed physical impairment, 25.2% cognitive impairment, and 12.9% psychological distress. **Fig 1** shows the distribution of impairments among the different domains.

We assessed the association of several potential predictors with the risk for PICS at 3 months adjusted for age and gender (**Table 2)**. Several factors were associated with PICS including baseline severity of illness scores (APACHE II: OR 1.07, 95%CI 1.02 to 1.12, p = 0.007 and SAPS II: OR 1.03, 95%CI 1.01 to 1.06, p = 0.006), intubation (OR 2.21, 95%CI 1.02 to 4.78, p = 0.043) and duration of intubation (in days) (OR 1.21, 95%CI 1 to 1.46, p = 0.046), length of ICU stay (in days) (OR 1.11, 95%CI 1.01 to 1.21, p = 0.022), functionality at discharge (poor mRS score: OR 4.35, 95%CI 1.7 to 11.1, p = 0.002 and CPC score: OR 3.39, 95%CI 1.46 to 7.88, p = 0.005), as well as work loss within the observed 3 months (OR 14.53, 95%CI 1.8 to 117.56, p = 0.012).

**Table 2:** Associations of predictor variables and post-intensive care syndrome at 3 months.

Factor		No PICS	PICS	OR (95%CI)	*p*	OR adjusted for age and gender (95%CI)	*p*
N		70	69				
**Sociodemographics**							
Age, median (IQR)		65.4 (58.6, 73.5)	61.1 (53.3, 73.3)	0.99 (0.97, 1.01)	0.46	*NA*	*NA*
Female, n (%)		9 (13%)	13 (19%)	1.57 (0.62, 3.96)	0.34	*NA*	*NA*
In partnership, n (%)		54 (79%)	55 (82%)	1.19 (0.5, 2.8)	0.69	1.25 (0.53, 2.99)	0.61
Children, n (%)		55 (79%)	59 (86%)	1.61 (0.67, 3.88)	0.29	1.73 (0.7, 4.28)	0.24
Highest education	School, n (%)	7 (12%)	5 (9%)	0.73 (0.22, 2.45)	0.61	0.95 (0.45, 2.02)	0.89
	Diploma/Apprenticeship, n (%)	38 (67%)	41 (76%)	1.58 (0.69, 3.62)	0.28	1.61 (0.7, 3.72)	0.27
	University, n (%)	12 (21%)	8 (15%)	0.65 (0.24, 1.75)	0.40	0.68 (0.25, 1.85)	0.45
Employed at baseline, n (%)		27 (41%)	34 (52%)	1.53 (0.77, 3.05)	0.22	1.69 (0.65, 4.36)	0.28
**Setting of cardiac arrest**							
Setting of cardiac arrest	At home	20 (29%)	18 (28%)	1.20 (0.66, 2.18)	0.55	1.27 (0.69, 2.35)	0.43
	In public	45 (65%)	39 (61%)				
	IHCA	4 (6%)	7 (11%)				
Observed cardiac arrest		63 (90%)	63 (91%)	1.16 (0.37, 3.67)	0.79	1.15 (0.37, 3.66)	0.80
	Bystander CPR	57 (81%)	55 (80%)	0.90 (0.39, 2.08)	0.80	0.89 (0.38, 2.09)	0.80
	Professional bystander	23 (55%)	14 (38%)	0.50 (0.20, 1.24)	0.13	0.48 (0.18, 1.31)	0.15
Initial rhythm	Ventricular tachycardia	4 (6%)	1 (1%)	1.05 (0.77, 1.44)	0.75	1.04 (0.77, 1.43)	0.77
	Ventricular fibrillation	52 (74%)	50 (74%)				
	Asystole	0 (0%)	6 (9%)				
	Pulseless electrical activity	6 (9%)	3 (4%)				
	Unknown	8 (11%)	8 (12%)				
No-flow (min), median (IQR)		0 (0, 4)	0 (0, 2)	1.01 (0.91, 1.12)	0.89	1.01 (0.91, 1.12)	0.91
Low-flow (min), median (IQR)		12 (10, 20)	12 (7, 23)	1.01 (0.99, 1.04)	0.36	1.01 (0.99, 1.04)	0.39
Time until ROSC (min), median (IQR)		15 (10, 21)	14 (8, 30)	1.01 (0.99, 1.04)	0.28	1.01 (0.99, 1.04)	0.32
Adrenaline	No adrenaline	38 (58%)	32 (53%)	1.19 (0.78, 1.82)	0.42	1.16 (0.75, 1.80)	0.50
	>0 and <3 mg	14 (22%)	12 (20%)				
	≥3 mg	13 (20%)	16 (27%)				
**Clinical scores at ICU arrival**							
APACHE II, median (IQR)		24 (17, 29)	26 (21, 31)	1.06 (1.02, 1.11)	**0.01**	1.07 (1.02, 1.12)	**0.007**
SAPS II, median (IQR)		55 (36, 65)	61 (51, 68)	1.03 (1.01, 1.05)	**0.01**	1.03 (1.01, 1.06)	**0.006**
GCS, median (IQR)		4 (3, 15)	4 (3, 8)	0.95 (0.89, 1.02)	0.17	0.96 (0.89, 1.02)	0.19
**Reason for OHCA**							
Coronary heart disease, n(%)		45 (66%)	47 (69%)	1.14 (0.56, 2.35)	0.71	1.24 (0.59, 2.6)	0.57
Rhythmogenic, n(%)		13 (19%)	9 (13%)	0.65 (0.26, 1.63)	0.35	0.62 (0.24, 1.58)	0.32
Other or unclear reason, n (%)		10 (15%)	12 (18%)	1.24 (0.5, 3.11)	0.64	1.15 (0.45, 2.92)	0.77
**Intensive care treatment**							
Intubation, n (%)		44 (63%)	55 (80%)	2.32 (1.08, 4.97)	**0.03**	2.21 (1.02, 4.78)	**0.04**
Total days of intubation, median (IQR)		2 (1, 2)	2 (2, 6)	1.21 (1.01, 1.45)	**0.04**	1.21 (1, 1.46)	**0.046**
Targeted temperature management (TTM), n (%)		34 (49%)	43 (62%)	1.75 (0.89, 3.44)	0.10	1.74 (0.86, 3.5)	0.12
Vasoactives, n (%)		56 (80%)	51 (74%)	0.71 (0.32, 1.57)	0.40	0.68 (0.31, 1.52)	0.35
Impella / IABP, n (%)		4 (6%)	5 (7%)	1.29 (0.33, 5.02)	0.71	1.21 (0.3, 4.84)	0.79
Sedatives, n (%)		58 (83%)	65 (94%)	3.36 (1.03, 11)	**0.05**	3.18 (0.97, 10.48)	0.06
Coronary angiography, n (%)		61 (87%)	63 (91%)	1.55 (0.52, 4.61)	0.43	1.59 (0.53, 4.79)	0.41
**Medical complications during ICU stay**							
Aspiration, n (%)		29 (41%)	28 (41%)	0.97 (0.49, 1.9)	0.92	0.99 (0.5, 1.96)	0.98
Pneumonia, n (%)		31 (44%)	33 (48%)	1.15 (0.59, 2.25)	0.68	1.19 (0.61, 2.35)	0.61
Hemorrhage, n (%)		5 (7%)	7 (10%)	1.47 (0.44, 4.87)	0.53	1.52 (0.46, 5.1)	0.50
Delirium, n (%)		25 (36%)	22 (32%)	0.84 (0.42, 1.7)	0.63	0.87 (0.43, 1.78)	0.71
Renal failure, n (%)		7 (10%)	11 (16%)	1.71 (0.62, 4.7)	0.30	1.7 (0.61, 4.74)	0.31
Seizure, n (%)		2 (3%)	7 (10%)	3.84 (0.77, 19.18)	0.10	4.13 (0.82, 20.84)	0.09
**Days in ICU, median (IQR)**		4 (2, 5)	4 (2, 7)	1.1 (1.02, 1.2)	**0.02**	1.11 (1.01, 1.21)	**0.02**
**Total days of hospital stay, median (IQR)**		12 (7, 16)	14 (9, 18)	1.03 (0.99, 1.07)	0.14	1.03 (0.99, 1.08)	0.13
Poor mRS score at ICU discharge, n (%)		7 (10%)	22 (33%)	4.33 (1.7, 11.01)	**0.002**	4.35 (1.7, 11.1)	**0.002**
Poor CPC score at ICU discharge, n (%)		10 (14%)	24 (36%)	3.29 (1.43, 7.6)	**0.01**	3.39 (1.46, 7.88)	**0.005**
**Follow-up on patients after 3 months**							
Rehabilitation	None, n (%)	24 (34%)	19 (28%)	0.73 (0.35, 1.5)	0.39	0.72 (0.35, 1.5)	0.38
	Up to 3 weeks, n (%)	25 (36%)	25 (36%)	1.02 (0.51, 2.04)	0.95	1.04 (0.52, 2.09)	0.91
	More than 3 weeks, n (%)	21 (30%)	25 (36%)	1.33 (0.65, 2.69)	0.44	1.31 (0.64, 2.67)	0.46
Working status	Still working, n (%)	26 (42%)	22 (36%)	0.78 (0.38, 1.61)	0.51	0.49 (0.18, 1.35)	0.17
	Work lost, n (%)	1 (2%)	11 (18%)	13.42 (1.67, 107.53)	**0.01**	14.53 (1.8, 117.56)	**0.01**
	No work prior to OHCA, n (%)	35 (56%)	28 (46%)	0.65 (0.32, 1.33)	0.24	0.51 (0.18, 1.46)	0.21

Note: Data are presented as n (%) or median (interquartile range). Abbreviations: IQR, interquartile range; ROSC, return to spontaneous circulation; IABP, intra-aortal balloon pump; mRS, modified Rankin Scale; CPC, Cerebral Performance Category; APACHE II, Acute Physiology And Chronic Health Evaluation Score II; SAPS II, Simplified Acute Physiology Score II.

### Secondary endpoint: PICS 12 months after hospitalization

**Fig 2:**
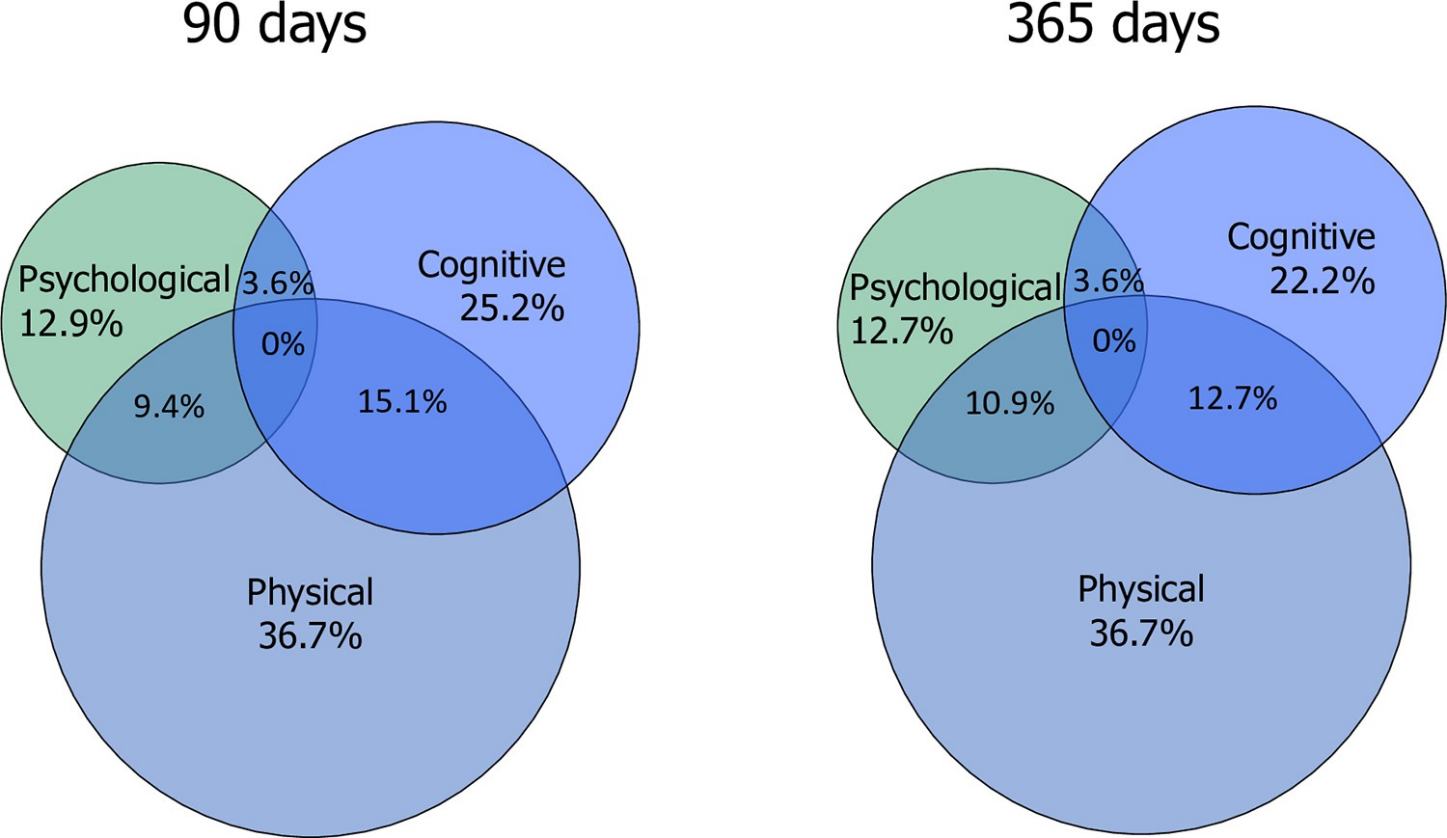
Co-occurrence of post-intensive care syndrome domains at 3 and 12 months. Note: Post-intensive care syndrome domains, i.e. physical, cognitive and psychological symptoms, and overlaps between the different domains.

Of 110 patients, 52 patients (47.3%) showed evidence of PICS after 12 months with 36.7% showing physical impairment, 22.2% cognitive impairment, and 12.7% psychological distress (**Fig 2**). We assessed potential predictors for PICS (**Table 3**) and found initial severity of illness scores (APACHE II: OR 1.08, 95%CI 1.02 to 1.14, p = 0.008) and functionality at discharge (poor mRS score: OR 3.97, 95%CI 1.42 to 11.12, p = 0.009; and CPC score: OR 3.22, 95%CI 1.29 to 8.04, p = 0.012) to be associated with PICS. In addition, risk for PICS was lower in patients not needing rehabilitation (OR 0.31, 95%CI 0.12 to 0.82, p = 0.019) and in turn increased with longer duration of the rehabilitation (in days) (OR 1.24, 95%CI 1.03 to 1.5, p = 0.027).

**Table 3:** Associations of predictor variables and post-intensive care syndrome at 12 months.

Factor		No PICS	PICS	OR (95% CI)	*p*	OR adjusted for age and gender (95% CI)	*p*
N		58	52				
**Sociodemographics**							
Age, median (IQR)		63.4 (54, 72.4)	61.1 (53.4, 74.5)	1.01 (0.98, 1.04)	0.49	*NA*	*NA*
Female, n (%)		8 (14%)	9 (17%)	1.31 (0.46, 3.69)	0.61	*NA*	*NA*
In partnership, n (%)		48 (84%)	42 (81%)	0.79 (0.29, 2.12)	0.64	0.78 (0.29, 2.11)	0.62
Children, n (%)		46 (79%)	46 (88%)	2 (0.69, 5.78)	0.20	1.85 (0.62, 5.5)	0.27
Highest education	School, n (%)	6 (12%)	6 (12%)	1.07 (0.32, 3.57)	0.91	1.17 (0.31, 4.34)	0.82
	Diploma/apprenticeship, n (%)	34 (65%)	38 (78%)	1.83 (0.76, 4.42)	0.18	1.79 (0.73, 4.36)	0.2
	University, n (%)	12 (23%)	5 (10%)	0.38 (0.12, 1.17)	0.09	0.37 (0.12, 1.15)	0.09
Employed at baseline, n (%)		29 (51%)	25 (49%)	0.93 (0.44, 1.98)	0.85	1.19 (0.45, 3.1)	0.73
**Setting of cardiac arrest**							
Setting of cardiac arrest	At home	14 (25%)	17 (33%)	0.83 (0.41, 1.64)	0.58	0.82 (0.41, 1.66)	0.59
	In public	39 (70%)	30 (59%)				
	IHCA	3 (5%)	4 (8%)				
Observed cardiac arrest		54 (93%)	52 (100%)	1	-	1	-
	Bystander CPR	46 (79%)	37 (71%)	0.64 (0.27, 1.54)	0.32	0.59 (0.24, 1.45)	0.25
	Professional bystander	15 (54%)	11 (61%)	1.36 (0.41, 4.54)	0.62	1.71 (0.42, 6.90)	0.45
Initial rhythm	Ventricular tachycardia	5 (9%)	2 (4%)	0.77 (0.51, 1.16)	0.21	0.77 (0.51, 1.17)	0.22
	Ventricular fibrillation	41 (71%)	40 (78%)				
	Asystole	1 (2%)	5 (10%)				
	Pulseless electrical activity	3 (5%)	4 (8%)				
	Unknown	8 (14%)	0 (0%)				
No-flow (min), median (IQR)		0 (0, 2)	0 (0, 2)	1.07 (0.96, 1.20)	0.24	1.08 (0.96, 1.21)	0.20
Low-flow (min), median (IQR)		11 (9, 17)	12 (6, 30)	1.02 (0.99, 1.05)	0.21	1.02 (0.99, 1.06)	0.13
Time until ROSC (min), median (IQR)		15 (10, 20)	20 (8, 30)	1.02 (0.99, 1.05)	0.13	1.03 (1, 1.06)	0.07
Adrenaline	No adrenaline	36 (69%)	20 (43%)	2.03 (1.19, 3.47)	**0.01**	2.30 (1.30, 4.09)	**0.004**
	>0 and <3 mg	10 (19%)	13 (28%)				
	≥3 mg	6 (12%)	13 (28%)				
**Clinical scores at ICU arrival**							
APACHE II, median (IQR)		24 (17, 28)	28 (22, 32)	1.08 (1.02, 1.14)	**0.01**	1.08 (1.02, 1.14)	**0.01**
SAPS II, median (IQR)		58 (39, 66)	60 (50, 70)	1.02 (1, 1.05)	0.11	1.02 (0.99, 1.05)	0.13
GCS, median (IQR)		5 (3, 15)	4 (3, 9)	0.97 (0.90, 1.04)	0.38	0.96 (0.89, 1.04)	0.30
**Reason for OHCA at ICU admission**							
Coronary heart disease, n (%)		38 (70%)	33 (63%)	0.73 (0.32, 1.65)	0.45	0.75 (0.32, 1.73)	0.49
Rhythmogenic, n (%)		10 (19%)	11 (21%)	1.18 (0.45, 3.07)	0.73	1.17 (0.44, 3.12)	0.75
Other or unclear reason, n (%)		6 (11%)	8 (15%)	1.45 (0.47, 4.52)	0.52	1.39 (0.44, 4.39)	0.58
**Intensive care treatment**							
Intubation, n (%)		37 (64%)	41 (79%)	2.12 (0.9, 4.97)	0.09	2.34 (0.97, 5.64)	0.06
Total days of intubation, median (IQR)		2 (1, 2)	2 (1, 6)	1.21 (0.98, 1.49)	0.08	1.25 (0.99, 1.58)	0.07
Targeted temperature management (TTM), n (%)		30 (52%)	31 (60%)	1.38 (0.65, 2.94)	0.41	1.61 (0.71, 3.63)	0.25
Vasoactives, n (%)		47 (81%)	38 (73%)	0.64 (0.26, 1.56)	0.32	0.65 (0.26, 1.59)	0.34
Impella / IABP, n (%)		5 (9%)	7 (13%)	1.65 (0.49, 5.55)	0.42	1.89 (0.54, 6.59)	0.32
Sedatives, n (%)		49 (84%)	48 (92%)	2.2 (0.64, 7.64)	0.21	2.28 (0.65, 8.02)	0.20
Coronary angiography, n (%)		50 (86%)	45 (87%)	1.03 (0.35, 3.06)	0.96	1.14 (0.37, 3.51)	0.82
**Medical complications during ICU stay**							
Aspiration, n (%)		25 (43%)	20 (38%)	0.83 (0.38, 1.77)	0.62	0.87 (0.4, 1.9)	0.73
Pneumonia, n (%)		28 (48%)	25 (48%)	0.99 (0.47, 2.1)	0.98	1.05 (0.49, 2.25)	0.90
Hemorrhage, n (%)		3 (5%)	8 (15%)	3.33 (0.83, 13.31)	0.09	3.36 (0.83, 13.54)	0.09
Delirium, n (%)		18 (31%)	18 (35%)	1.18 (0.53, 2.61)	0.69	1.18 (0.53, 2.64)	0.68
Renal failure, n (%)		5 (9%)	10 (19%)	2.52 (0.8, 7.95)	0.11	2.46 (0.77, 7.81)	0.13
Seizure, n (%)		2 (3%)	4 (8%)	2.33 (0.41, 13.3)	0.34	2.63 (0.45, 15.27)	0.28
**Days in ICU, median (IQR)**		4 (2, 8)	4.5 (2, 7)	1.03 (0.96, 1.11)	0.39	1.05 (0.97, 1.13)	0.27
**Total days of hospital stay, median (IQR)**		13 (8, 16)	14 (7, 21)	1.03 (0.98, 1.07)	0.22	1.03 (0.98, 1.07)	0.20
Poor mRS score at ICU discharge, n (%)		6 (11%)	17 (33%)	4.05 (1.45, 11.29)	**0.01**	3.97 (1.42, 11.12)	**0.01**
Poor CPC score at ICU discharge, n (%)		9 (16%)	20 (38%)	3.26 (1.32, 8.08)	**0.01**	3.22 (1.29, 8.04)	**0.01**
**Follow-up on patients after 3 months**							
Rehabilitation	None	19 (33%)	7 (13%)	0.32 (0.12, 0.84)	**0.02**	0.31 (0.12, 0.82)	**0.02**
	Up to 3 weeks	21 (36%)	16 (31%)	0.78 (0.35, 1.74)	0.55	0.79 (0.36, 1.77)	0.57
	More than 3 weeks	18 (31%)	29 (56%)	2.8 (1.28, 6.11)	**0.01**	2.88 (1.3, 6.38)	**0.01**
Working status	Still working	26 (48%)	17 (36%)	0.61 (0.27, 1.36)	0.23	0.69 (0.23, 2.01)	0.49
	Work lost	3 (6%)	7 (15%)	2.98 (0.72, 12.24)	0.13	3.07 (0.74, 12.82)	0.12
	No work prior to OHCA	25 (46%)	23 (49%)	1.11 (0.51, 2.43)	0.79	0.72 (0.24, 2.11)	0.55

Note: Data are presented as n (%) or median (interquartile range). Abbreviations: IQR, interquartile range; ROSC, return to spontaneous circulation; IABP, intra-aortal balloon pump; mRS, modified Rankin Scale; CPC, Cerebral Performance Category; APACHE II, Acute Physiology And Chronic Health Evaluation Score II; SAPS II, Simplified Acute Physiology Score II.

**Fig 3:**
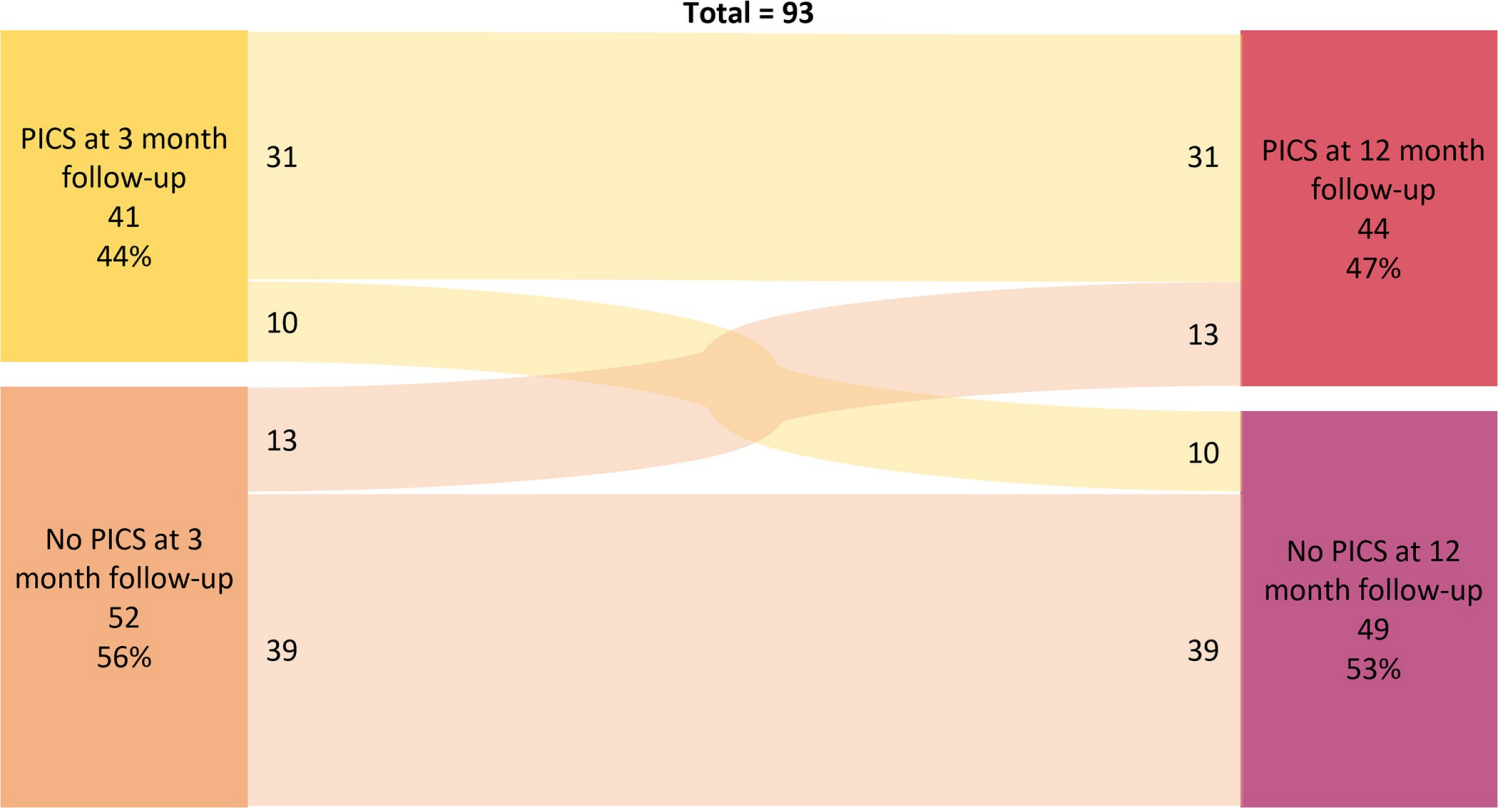
Sankey diagram of occurrence of PICS or no PICS at 3 and 12 months.

We also investigated, in the 93 patients that were assessed at both time points, whether PICS at 3-month would persist after 12-month. Results stratified according to PICS at both time points are shown in **Fig 3.** Chi-square test between PICS at 3 and 12 months was significant, X^2^(1, N = 93) = 23.6, p < .001. Further, we investigated how the different domains of PICS were inter-correlated by calculation of a correlation matrix at 3- and 12-month as shown in **Table 4**. Correlations between PICS domains at 3-month follow-up showed significant correlations between the physical and psychological domain and between the physical and cognitive domain. Similar results were found at 12-month follow-up.

**Table 4:** Correlation matrix of physical, cognitive and psychological domain at 3- and 12-month follow-up.

PICS domains at 3 months				PICS domains at 12 months		
	Physical domain	Cognitive domain	Psychological domain	Physical domain	Cognitive domain	Psychological domain
Physical domain	-	-	.	-	-	-
Cognitive domain	0.28, p<0.001	-	-	0.25, p<0.01	-	-
Psychological domain	0.28, p<0.001	0.02, p = 0.79	-	0.39, p<0.001	0.06, p = 0.55	-

Note: Data reported in Pearson correlation coefficient r.

## Discussion

In this prospective observational cohort study, we found that nearly half of our OHCA survivors suffered from long-term health impairments after their ICU stay. One in three patients showed physical impairments, one in four had cognitive impairments, and one in eight patients psychological distress. These findings were comparable at 3 and 12 months following cardiac arrest with similar percentages overall and within domains. We found weak, yet significant correlations between domains except for the psychological and cognitive domain. Furthermore, several baseline predictors were identified as potential risk factors.

This study has several important implications. First, the prevalence of PICS found in our cohort of OHCA surviors is comparable to other cohorts of general ICU patients at 3 and 12 months [8]. Yet, there are differences in the distribution among PICS domains. We found similar rates of physical impairments of almost 40% in our cohort compared to studies from the general ICU patient population [32]. In contrast to other reports showing an improvement in self-assessed health at long-term [11,12], our cohort was fairly stable within the 12 months of investigation. Furthermore, we found cognitive impairments in 25% and 22% of patients at 3 and 12 months. Importantly, these numbers may be influenced by the instrument used for assessment: objective assessments of cognitive impairment have found higher prevalences compared to subjective assessments [13]. We used a subjective instrument for assessing cognitive impairment [33], which may explain the lower risks, which is again in line with other reports [14]. Also, one third to nearly half of ICU patients have been found to suffer from mental health issues [34,35]. For OHCA patients, previous reports ranged between 14% to 45% for depression and from 13% to 61% for anxiety, again dependent on the instrument and cut-offs used [15]. Our findings of 13% at both time points are thus in the lower range of these studies [15].

Second, several clinical and psychosocial factors were related to developing PICS at 3 months including severity of illness, adrenaline, intubation, functionality at discharge and work loss within 3 months post-discharge. These risk factors, however, are challenging to modify. Prolonged mechanical ventilation or deep sedation have previously been found to aggravate symptoms of PICS [36,37]. Thus, daily stop of anesthetics to avoid oversedation, early weaning strategies and use of lower sedative drug doses have become an important goal in any ICU patient care [37,38]. Additionally, we found that the need for rehabilitation and prolonged rehabilitation were associated with PICS 12 months after OHCA. Our data indicate that during rehabilitation, screening for PICS could help identify high-risk patients needing medical and psychological support, which in turn may reduce their risk in the long term. This may be important not only for the individual patient but also on a larger economic and social level [39].

Similarly, cognitive impairment at discharge assessed by the mRS and CPC score was associated with PICS 3 and 12 months following OHCA. This association may at least partially be explained by the cognitive impairments we had already found at baseline persisting in the long-term. This is in line with research, showing that most recovery of cognitive function in ICU patients occurred within the first 3 months with only little improvements after 12 months [40]. Thus, measures of cognitive functioning may be useful in screening patients to predict long-term PICS early on.

Interestingly, no patients had impairments in all three PICS domains at neither time point of assessment. This is in line with other results in general ICU patients: Marra et al. found a 56% prevalence of PICS-related complaints when considering one or more domains, but a much lower prevalence when complaints in all three domains were considered (i.e., 4% after 12 months) [8]. Concerning the co-occurrence of the different PICS domains, we found weak, yet significant correlations between domains except between the psychological and cognitive domains. This coincides with findings in OHCA patients that show health-related quality of life to be associated with cognitive impairments [14,41], as well as with psychological distress [14,16], yet finding mixed results in associations between psychological distress and cognitive impairment [16,42]. Possibly, PICS in OHCA patients falls into two subgroups: physically and cognitively impaired patients, or physically impaired and psychologically distressed patients. However, this hypothesis must be validated in future research.

Our findings suggest that PICS at 3 months is highly predictive for PICS after 12 months. At the same time, our data show that 11 patients newly developed physical impairment, 6 developed cognitive impairment and 8 patients developed psychological distress at twelve-month follow-up. Research shows levels of psychological distress and self-assessed health to improve among OHCA survivors in the long term [11,12], yet only minor improvements have been found in cognitive performance from 3 to 12 months [14]. However, these results are average findings and are comparable to our percentual stability of PICS impairments over time. Yet to the best of our knowledge, no analysis has assessed the course of symptoms as fine-grained as our study, therefore, intraindividual trajectories in other studies remain unclear. Possibly, due to patients’ self-report as only information, subjective health impairment may become more visible in everyday life over time.

This trial is strengthened by the prospective and consecutive inclusion of study patients. Yet, it also has several limitations. As an observational study, the results are in need for interventional research to prove causality. Also, due to the sample size the power of the study is limited. Further, 83% of the study cohort are men, however, we adjusted for gender in the multivariable model to control for possible confounding. Also, patient outcomes were assessed subjectively, therefore outcomes might differ to objective outcome measures. Further, as several patients were not reachable at either 3- or 12-month follow-up, only a subgroup could be assessed for PICS trajectories over time. Also, as a single-center study, there is a lack of generalizability to other institutions and countries. Therefore, multicenter and multinational studies are necessary to validate our findings. Further, since no single definition of PICS exists, comparability with other study findings is limited. We do not expect biased results by the telephone assessment, as no difference has been found between face-to-face and telephone self-report measures [43]. Within this hypothesis generating study, we aimed to understand the possible associations of baseline factors and long-term risk for PICS. Because there is insufficient literature on this topic, we did not preselect variables but present the full list of predictors and due to the limited number of events, we adjusted the analysis only for age and gender. The high number of tests makes type II error possible and prospective validation is needed in an independent cohort. Finally, in our analysis acute physiology parameters wane in importance as time from OHCA passes, but mRS and CPC continue to dominate the associations. This may be indeed specific to the population of OHCA patients with brain injury and may differ in other ICU populations. However, more data is needed to better understand the influence on brain injury on long-term risk for PICS.

## Conclusions

With a growing number of patients surviving their ICU stay after an OHCA and nearly half of all OHCA survivors displaying evidence of PICS up to one year after ICU admission, appropriate screening and management is necessary to minimize the risk for PICS and to meet the increased need for its treatment. Future studies should evaluate whether early identification of these patients enables preventive strategies.

## Supporting information

S1 Database (XLSX)
